# The Role of Preoperative Chronic Hypertension in Neurocognitive Decline after Cardiac Surgery: A Retrospective Cohort Study

**DOI:** 10.21470/1678-9741-2023-0470

**Published:** 2025-02-05

**Authors:** Madigan E. Stanley, Ronald K. Phillips III, Jun Feng, Guangbin Shi, Shawn Kant, Nicholas C. Sellke, Neel R. Sodha, Afshin Ehsan, Frank W. Sellke

**Affiliations:** 1 Division of Cardiothoracic Surgery, Rhode Island Hospital, Alpert Medical School of Brown University, Providence, Rhode Island, United States of America

**Keywords:** Cardiac Surgery, Neurocognitive Decline, Cognitive Dysfunction, Postoperative Cognitive Complications, Neuropsychological Tests

## Abstract

**Introduction:**

Patients frequently experience transient postoperative neurocognitive decline (NCD) after cardiac surgery with cardiopulmonary bypass. The goal of this study is to describe preoperative high blood pressure as a risk factor for NCD and use genomic expression to uncover its contribution to the pathophysiology of NCD.

**Methods:**

This is a retrospective analysis of cohort study at a single academic center. Patients undergoing cardiac surgery with the use of cardiopulmonary bypass were administered the Repeatable Battery for the Assessment of Neuropsychological Status (RBANS) preoperatively, at postoperative day four, and four weeks postoperatively. Electronic medical records were reviewed for all recorded blood pressure from the year preceding surgery and intraoperative blood pressures. Blood samples were collected six hours preoperatively and six hours postoperatively to assess messenger ribonucleic acid expression.

**Results:**

Eighty-seven patients completed postoperative day four testing, of whom thirty-seven experienced NCD (42.5%). Chronically elevated systolic blood pressure over the year preceding surgery was correlated with greater negative change in RBANS score at postoperative day four (*P*=0.03). Upon genomic analysis, macrophage markers were upregulated preoperatively, and anti-inflammatory and neuroprotective genes were downregulated postoperatively among patients who had a mean systolic blood pressure ≥ 130 mmHg.

**Conclusion:**

Chronic exposure to elevated preoperative systolic blood pressure may increase the risk of NCD. The contributing role of preoperative hypertension in NCD may be partly explained by reduced attenuation of oxidative stress, increased inflammation, and reduced neuroprotection and heme metabolism postoperatively. This must be considered when assessing patient risks for cardiac surgery.

## INTRODUCTION

Cardiopulmonary bypass (CPB) is used to maintain perfusion and oxygenation in a patient undergoing cardiac surgery during cessation of the heartbeat^[1]^. Despite its common use in cardiac surgery, CPB has been associated with neurocognitive decline (NCD) in up to 70% of patients^[2]^. Postoperative NCD encompasses cognitive symptoms which are temporally related to surgery and require pre and postoperative cognitive assessment to diagnose. Many of the neurologic symptoms associated with NCD are transient and involve deficits in cognition, memory, awareness, and attention, as well as confusion and behavioral changes^[2]^. NCD after surgery has additionally been linked to future accelerated cognitive decline^[3,4]^. This suggests that NCD has a biphasic clinical impact on patients: an acute phase that patients recover from in about two months, and a chronic phase leading to poorer long-term cognitive function^[3,4]^.

The mechanism of cognitive injury postoperatively is not entirely clear, and its complex pathophysiology may explain the lack of surgical outcome improvement. Our lab and others have contributed to an explanation of NCD, which involves systemic inflammation caused by CPB and cardiac surgery: cytokine release, leukocyte activation, oxidative stress, complement, lack of cerebral perfusion, and more^[5,6,7,8]^.

Hypertension has a role in chronic systemic inflammation^[9]^. Higher cumulative lifetime blood pressure exposure has been independently associated with cognitive decline, dementia, and all-cause mortality in adults over 49 years old^[10]^. However, preoperative exposure to chronic hypertension has yet to be evidently established as a risk factor for NCD after cardiac surgery. This preliminary study seeks to assess preoperative hypertension as a risk factor for NCD and to explain its role in the pathogenesis of NCD after cardiac surgery using genomics. We hypothesized that patients with prolonged exposure to preoperative hypertension would have increased incidence of postoperative NCD.

## METHODS

### Patient Enrollment

This is a single-institution, retrospective study of prospectively enrolled cohort of patients between 2017 to 2022. There was a brief break in enrollment due to research staffing. Patients over eighteen years of age undergoing coronary artery bypass grafting, aortic valve replacement, mitral valve repair or replacement, or combined procedure utilizing CPB were eligible for enrollment. Consecutive patients were approached for enrollment during their preoperative outpatient appointment or while hospitalized with surgery planned during the same admission. Patients were included if they consented to the study and completed both the preoperative and postoperative day four (POD4) Repeatable Battery for the Assessment of Neuropsychological Status (RBANS) assessment. Exclusion criteria included patients whose primary language is not English, patients with hepatic disease, stroke within one year of surgery, baseline neurologic deficits or dementia, chronic renal failure, carotid stenosis of over 70%, heavy aortic calcification, or impaired vision/blindness. Patients were also excluded if their procedures involved the aortic arch/root or carotid arteries. This study was approved by the Institutional Review Board at Lifespan (Project ID#225612-69; initial approval date 03/10/2010).

Written consent was obtained from each patient to include their information in this publication.

### Clinical Data Collection

Our study aim was to measure actual patient exposure to high blood pressure preoperatively, so chart review was completed for each patient documenting all systolic and diastolic blood pressures recorded at each visit in the electronic medical record for the year preceding surgery. Systolic blood pressure (SBP) was used to measure high blood pressure exposure and divide patients into exposure groups. Normotensive is considered patients with an average SBP and maximum SBP < 130 mmHg. This cutoff was chosen in accordance with American College of Cardiology and American Heart Association guidelines. Moderate chronic hypertension was considered patients with an average SBP between 130 and 145 mmHg. High chronic hypertension was considered patients with an average SBP > 145 mmHg. Intermittent hypertension was defined as patients with an average SBP < 130 mmHg, but with maximum SBP recordings > 130 mmHG. Actual patient exposure to high blood pressures preoperatively was decided to be the best represented risk level rather than history of hypertension. Normotensive criteria were achieved regardless of previous diagnosis of hypertension or home medications, encompassing patients who successfully maintain optimal blood pressures through pharmaceuticals and/or lifestyle despite a hypertension diagnosis.

### Surgical Technique

Standard methods were used during cardiac surgery including induction of general anesthesia, invasive monitoring, midline sternotomy, and systemic heparinization. The CPB circuit utilized a Medtronic Affinity integrated hollow fiber oxygenator/cardiotomy reservoir with trillium coating (Medtronic, Minneapolis, Minnesota, United States of America) and an arterial 38 mg-filter with trillium coating (Medtronic Affinity). The cardioplegia perfusion system included trillium coating (Medtronic Myotherm 4:1 system). Moderate hypothermia to temperatures between 32°C and 34°C was used while on CPB. A hypothermic blood-based cardioplegia solution (8°C, 4:1 mixture of oxygenated blood and hyperkalemic crystalloid solution) (CAPS, Lanham, Maryland, United States of America) was used. An initial 650 to 1000 mL of hyperkalemic (K+ 25 mmol/L) cardioplegia solution was delivered antegrade into the aortic root, followed by 200-500 mL of cardioplegia solution (K+ 8 mmol/L) every 15-20 minutes until cross-clamp was removed.

Anesthetic protocol varies between anesthesiologists and patients, but there is a standard methodology of anesthetic management during cardiac surgery. Patients are premedicated with midazolam or dexmedetomidine. Anesthesia is induced with titrated propofol, rocuronium is given for muscle relaxation, and isoflurane is used for maintenance. Dosing during surgery and potential addition of nitroglycerin depend solely on hemodynamic response to the standard regimen, varying between patients. During CPB, a target mean arterial pressure (MAP) maintained is between 50 mmHg and 70 mmHg. Each patient’s MAP during CPB fell within this range. Cerebral oximetry is used in many cases and monitored by the cardiac anesthesiologists. Additionally, most patients receive a small dose of hydromorphone, as well as dexmedetomidine for transitioning to intensive care unit (ICU) sedation.

### Postoperative Care

All patients remain intubated at the end of the case and are admitted to the cardiothoracic ICU postoperatively. Our institutional goal is to extubate within four hours postoperatively unless there is a specific need for continued ventilatory support. While intubated, patients receive intermittent intravenous opioids (fentanyl or hydromorphone) for pain control and intravenous sedation. Patients are quickly transitioned to oral opioid medications once started on a diet. Appropriate home medications are resumed as early as clinically tolerated including daily home psychiatric medications. Melatonin is occasionally prescribed as a sleep aid if needed. Prior to completing POD4 RBANS testing, patients were briefly screened for orientation.

### Neurocognitive Testing

Patient cognitive function was tested using RBANS (Pearson Clinical Assessments, Bloomington, Minnesota, United States of America). RBANS measures immediate and delayed memory, language, attention, and visuospatial skills. Following the suggestions of previous literature, the battery was administered three times^[11,12]^. The first administration, RBANS form A, was during the preoperative outpatient appointment or preoperative inpatient admission. The second administration, RBANS form B, was prior to discharge at POD4 to avoid the effects of emergence delirium or anesthesia. RBANS form C was administered one month postoperatively in patients willing to be examined at that time. Three iterations of the RBANS test were used to diminish test/retest and familiarity effects. Scores were scaled based on normative data published by the manufacturer with a fiftieth percentile score of 100 and a normal distribution score range of 40-160. Patients who yielded at least an eight-point RBANS score deficit postoperatively experienced NCD with a 90% confidence interval according to current literature^[12]^.

### Blood and Genomic Analysis

Peripheral intravenous blood was collected immediately before surgery and at six hours postoperatively. The blood collected was stored in ethylenediaminetetraacetic acid and Pax gene tubes which were stored and transferred on ice. The tubes were then centrifuged at 4°C at 2000 rpm for 20 minutes, and supernatant plasma was transferred to 1 mL aliquots and stored at -80°C. Gene expression and protein pathway association were performed by assessing messenger ribonucleic acid (mRNA) as a marker of gene expression using Applied Biosystems™ Clariom™ D Pico Assay (Thermo Fisher Scientific; Waltham, Massachusetts, United States of America) as indicated by the manufacturer. Upon microarray analysis, we chose to examine genes with both a significantly changed expression (*P*<0.05), measured by comparing the mean expression of each gene between patient groups, as well as a fold-change of ≥ 2.

### Statistical Analysis

Continuous variables were compared using unpaired *t*-tests with Welch’s correction. Categorical variables were compared using Chisquare or Fisher’s exact test. The interaction between categorical variable and multiple quantitative variables was examined using a one-way analysis of variance (ANOVA). Simple linear regression was used to examine the relationship between two continuous variables, blood pressure measurements, and RBANs scores, using a 95% confidence interval. The overall regression is reported including R^2^, degrees of freedom, and *P*-value. A *P*-value < 0.05 was considered to indicate statistical significance for all tests. Patient randomization was not performed because patients were compared against themselves pre and postoperatively. Statistical analysis was performed using GraphPad Prism 9.50 (San Diego, California, United States of America).

## RESULTS

### Hypertension and Neurocognitive Decline

Ninety-three patients completed written informed consent and preoperative neurocognitive testing. Eighty-seven patients completed POD4 neurocognitive testing and are thus included in the following analysis. Thirty-seven (42.5%) patients experienced NCD on POD4. Most patients (86%) had a diagnosis of hypertension listed in their medical record. Twenty (23.0%) patients met criteria for normotensive, 19 (21.8%) patients met criteria for intermittent hypertension, 33 patients (37.9%) met criteria for moderate chronic hypertension, and 15 patients (17.2%) met criteria for high chronic hypertension. The demographic and clinical characteristics of the patient cohort can be found in Table 1. The average age was 66.9 years (standard deviation [SD]=7.8), and 56 (64.4%) patients were male. The average preoperative RBANS total scaled score was 90.1 which is equivalent to the 25^th^ percentile based on published normative data. The SD of the preoperative RBANS total scaled score was 11.6, suggesting a range from the 8^th^ to 53^rd^ percentile based on published normative data.

**Table 1:** Patient demographics and clinical characteristics.

Demographics	N = 87
Sex (male), n (%)	56 (64.4)
Age, years, mean (SD)	66.9 (7.8)
Preoperative RBANS total scaled score, mean (SD)	90.1 (11.6)
**Clinical Characteristics**	
Hypertension, n (%)	75 (86.2)
Diabetes, n (%)	33 (37.9)
Body mass index, mean (SD)	30.9 (6.1)
Preoperative hemoglobin A1c, %, mean (SD)	6.38 (1.3)
Preoperative hematocrit, %, mean (SD)	41.45 (4.9)
Preoperative platelets, ×10^9/L, mean (SD)	221.7 (54.1)
Preoperative creatinine, mg/dL, mean (SD)	0.97 (0.35)
**Type of Surgery**	
CABG only	42 (48.3)
CABG + valve	15 (17.2)
AVR only	20 (23.0)
MVR only	5 (5.7)
Other combination	5 (5.7)
**Perioperative Outcomes**	
Cardiopulmonary bypass time, minutes, mean (SD)	118 (54)
ICU length of stay, days, median (IQR)	3.00 (2.00-4.00)
Hospital length of stay, days, median (IQR)	7.00 (6.00-8.00)

AVR=aortic valve replacement; CABG=coronary artery bypass grafting; ICU=intensive care unit; IQR=interquartile range; MVR=mitral valve repair or replacement; RBANS=Repeatable Battery for the Assessment of Neuropsychological Status; SD=standard deviation

**Fig. 1A:**
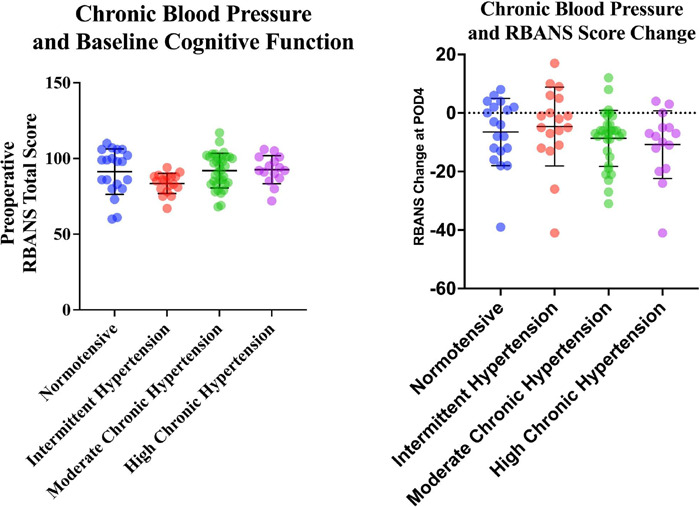
Relationship between chronic blood pressure and baseline cognitive function. A one-way analysis of variance (ANOVA) was performed to compare the effect of chronic systolic blood pressure on baseline Repeatable Battery for the Assessment of Neuropsychological Status (RBANS) total scaled score. The one-way ANOVA revealed that there was not a statistically significant difference in RBANS total score between at least two groups (F [3,83] = 2.67, P=0.05). Normotensive = mean and maximum systolic blood pressure < 130 mmHg. Intermittent hypertension = mean systolic blood pressure < 130 mmHg, but maximum systolic blood pressure > 130 mmHg. Moderate chronic hypertension = mean systolic blood pressure between 130 and 145 mmHg. High chronic hypertension = mean systolic blood pressure > 145 mmHg.

A one-way ANOVA was performed to compare the effect of chronic SBP on preoperative RBANS total scaled score. The one-way ANOVA revealed that there was not a statistically significant difference in RBANS score between at least two groups (F [3,83] = 2.67, *P*=0.05) (Figure 1). Follow-up testing using *t*-tests with Welch’s correction demonstrated a significant difference in preoperative RBANS total scaled score between patients in the normotensive group (M = 91.3, SD = 15.0) and the intermittent hypertension group (M = 83.5, SD = 6.7, *P*=0.04).

**Fig. 1B** Relationship between chronic blood pressure and postoperative day 4 (POD4) cognitive function. A one-way analysis of variance (ANOVA) was performed to compare the effect of chronic systolic blood pressure on change in Repeatable Battery for the Assessment of Neuropsychological Status (RBANS) total scaled score from preoperative to POD4. The one-way ANOVA revealed that there was not a statistically significant difference in RBANS total score between at least two groups (F [3,83] = 0.97, P=0.4). Normotensive = mean and maximum systolic blood pressure < 130 mmHg. Intermittent hypertension = mean systolic blood pressure < 130 mmHg, but maximum systolic blood pressure > 130 mmHg. Moderate chronic hypertension = mean systolic blood pressure between 130 and 145 mmHg. High chronic hypertension = mean systolic blood pressure > 145 mmHg.

**Fig. 2:**
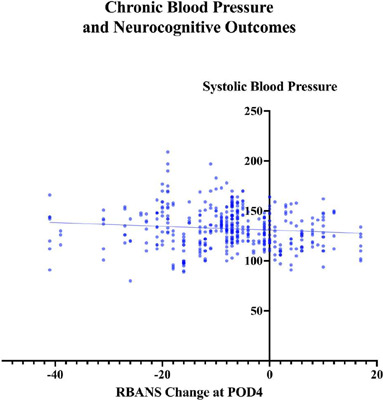
Interaction between chronic hypertension and neurocognitive outcomes. Linear regression was used to examine the relationship between systolic blood pressure from the year preceding surgery and the change in Repeatable Battery for the Assessment of Neuropsychological Status (RBANS) score on postoperative day four (POD4). This graphic includes all blood pressure measurements, both outpatient and preoperative, for all 87 patients. The overall regression was statistically significant (R2 = 0.01, F [1, 439] = 4.64, P=0.03).

**Fig. 3:**
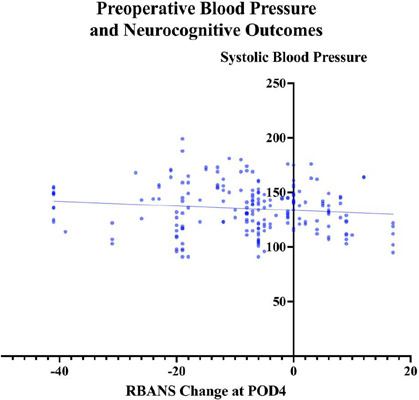
Interaction between preoperative blood pressure and neurocognitive outcomes. Linear regression was used to examine the relationship between preoperative systolic blood and the change in Repeatable Battery for the Assessment of Neuropsychological Status (RBANS) score on postoperative day four (POD4). Preoperative blood pressure measured either overnight for admitted patients or in the preoperative care unit. There was a trend towards a relationship (R2 = 0.01, F [1, 199] = 2.822, P=0.09).

Linear regression was used to examine the relationship between SBP over the year preceding surgery and the change in RBANS total scaled score on POD4. The overall regression was statistically significant (R2 = 0.01, F [1, 439] = 4.64, *P*=0.03) (Figure 2). Linear regression was also used to examine the relationship between preoperative systolic blood and the change in RBANS score on POD4. Preoperative blood pressure was measured either overnight for admitted patients or in the preoperative care unit. There was possibly a trend towards a relationship, although not statistically significant (R2 = 0.01, F [1, 199] = 2.82, *P*=0.09) (Figure 3).

There was not a statistically significant correlation between MAP on CPB and magnitude of NCD (F [1,81] = 2.3, *P*=2.820.13). Previous literature generally suggests a negative relationship between MAP on bypass and cognitive outcomes. There was no relationship between central venous pressure and change in RBANS score. There was no statistically significant relationship between higher diastolic blood pressure over the year preceding surgery and greater negative RBANS total score change at POD4 (*P*=0.15).

Fifty-nine (67.8%) patients completed testing at one-month postoperatively. Of those who completed one-month follow-up testing, eight patients (13.6%) demonstrated NCD. There was no correlation between SBP over the year preceding surgery and change in RBANS total score at one-month.

### Gene Expression and Pathways

Pre and postoperative samples were successfully obtained from 26 patients for gene expression analysis. Patients with a mean SBP > 130 mmHg were labeled as hypertensive (N=15), and those with a mean SBP < 130 mmHg were labeled as normotensive (N=11). Patients are not separated out according to their neurocognitive testing results. Hypertensive patients had significantly upregulated macrophage markers, cluster of differentiation (CD) 68 and CD74, present preoperatively (*P*<0.001). Genes involved in oxidative stress attenuation (nuclear factor erythroid 2-related factor 2, glutathione), anti-inflammation (interleukin [IL]-10 signaling pathway), and heme metabolism (alpha hemoglobin stabilizing protein, ferrochelatase, hydroxymethylbilane synthase) were significantly downregulated in these patients postoperatively. There were also genetic markers associated with lack of neuronal protection as well as neurodegeneration. These key findings are displayed in Table 2^[13,14,15,16,17,18,19,20,21,22]^. Among the subset of patients who had gene expression analysis, the mean CPB time was longer in the hypertensive group.

**Table 2:** Genomic expression analysis for subset of 26 patients. Notable genomic expression changes among hypertensive patients postoperatively as compared to normotensive patients. Pathway associations are determined by the Applied Biosystems™ Clariom™ D Pico Assay’s genomic database or by literature search.

Function	Genes	Pathway	*P*-value	Fold Change
**Inflammation**	BLVRB	NRF2 antioxidant synthesis pathway	0.02	-2.8
		IL-10 anti-inflammatory signaling pathway		
	GCLC	NRF2 antioxidant synthesis pathway	0.03	-2.3
		Glutathione biosynthesis		
		NRF2-ARE antioxidant and anti-inflammatory pathway, mitochondrial protection		
		Prevention of ferroptosis		
		Oxidative stress attenuation		
	JCHAIN	Antibodies	0.03	7.06
	NUDT4	Prevention of DNA damage	0.03	-2.5
**Neuronal Protection**	ANK1	Reduced expression associated with Alzheimer’s disease^[18]^	0.03	-2.3
	ARHGAP11B	Cortical development^[21]^	0.01	-2.0
	DEFA1, DEFA1B, DEFA3	Increased expression associated with Alzheimer’s disease^[19]^	0.01	2.7
	FBXO7	Protection from iron damage and neurodegeneration^[15]^	0.04	-2.5
	MIR548AI	microRNA marker in post-stroke depression^[22]^	0.04	-5.3
	PSMF1	Proteasome function, associated with Alzheimer’s disease^[20]^	0.02	-2.66
	PRDX2	Protection of neurons against beta amyloid^[16]^	0.02	-2.4
	SLC14A1	Neuroprotection from urea^[17]^	0.02	-2.9
	TMOD1	Dendrite modulation^[23]^	0.04	-2.6
**Heme metabolism**	AHSP	Hemoglobin stabilization, neuronal aging^[14]^	0.04	-5.6
	HMBS	Mitochondrial function, heme synthesis, neuronal aging^[14]^	0.04	-5.2
	FECH	Heme synthesis, neuronal aging^[14]^	0.03	-4.3

DNA=deoxyribonucleic acid; IL=interleukin; RNA=ribonucleic acid; NRF2=nuclear factor erythroid 2-related factor 2; NFR2-ARE=nuclear factor erythroid 2-related factor 2/antioxidant response element

## DISCUSSION

This pilot study suggests that chronically high SBP preoperatively is a risk factor for NCD and uncovers a potential mechanism of its contribution to the pathogenesis of NCD. Patients with higher preoperative SBP exhibited a larger decline from baseline RBANS to POD4 testing. Fortunately, nearly all patients recovered their neurocognitive function within one month of surgery, which is consistent with previous literature^[3,4]^. Linear regression indicates that the magnitude of NCD experienced may be related to the magnitude of preoperative hypertension^[9]^. Our genomic analyses indicate that the mechanism by which poor blood pressure control prior to surgery contributes to NCD may involve increased neuroinflammatory activity, interrupted heme metabolism, and a reduced ability to protect neurons and to attenuate inflammation and oxidative stress. This should be considered for risk assessment and patient management in the context of cardiac surgery. While poor blood pressure control preoperatively was associated with the neurologic injury markers seen in Table 2, the relationship between preoperative blood pressure and long-term cognitive decline cannot be determined by this study.

Differences in cerebral blood flow during and after cardiac surgery with CPB likely plays a role in the differential effect of surgery in patients with hypertension and with normal blood pressure prior to surgery. Hypertension alters the structure and function of the cerebral microcirculation, promotes microvascular rarefaction, inflammation, and neurovascular uncoupling, and may disrupt the blood–brain barrier^[23]^. Marked alterations in vasomotor regulation have been documented in the brain and various other organs after surgery utilizing CPB both *in vitro*^[24,25,26,27]^ and *in vivo*^[28]^. These changes in vasomotor regulation and cellular signaling may affect patients differently depending on their preoperative hypertensive state and factors such as the age of the patient^[5]^. Measures of absolute cerebral perfusion and/or oxygenation during and after cardiac surgery should be explored in future studies.

CPB, surgical trauma, and reperfusion injur y are well known inducers of a systemic inflammatory response in the body as observed by heightened complement activation, cytokine levels, free radicals, adhesion molecules, and NF-kB activation — ultimately resulting in tissue damage, cell death, and edema^[29]^. Amongst the systemic effects of CPB, our lab has sought to describe NCD after CPB in detail. Previous research has begun to determine the role of genomic expression involved in NCD after cardiac surgery. Preoperatively, patients who develop NCD significantly expressed genes involved in inflammation, cell death, and neurologic dysfunction, with a particular emphasis on T-cell activation and signaling^[7]^. Postoperatively, the pattern of gene expression is not as clear or uniform with regards to NCD. One study demonstrated that those who experience NCD have upregulation of pathways involved in inflammation, cell death, and neurologic dysfunction persisting at both six hours postoperatively and at POD4^[7]^. The genes involved in neurologic dysfunction are associated with amyloid plaques, decreased brain volume, Alzheimer’s disease, axonal and microtubule function, synaptic vesicle docking and neurotransmitter release, and neuronal dysfunction^[7]^. Another study revealed a contradictory decrease in expression of T-cell activators, inflammation, antigen presentation, and adhesion molecules among NCD patients, with an increased regulation in genes involved in blood coagulability^[6]^. While the picture is unclear, the current literature supports the possibility that preoperative genomic disposition to NCD is associated with an accentuated postoperative inflammatory response. Our study agrees with this possibility.

Multiple biomarker changes have been importantly linked to NCD. Previous research has demonstrated that NCD is associated with increased chemokines responsible for inducing leukocyte migration and blood brain barrier dysfunction^[30]^. NCD is also associated with the cytokines C-reactive protein (CRP), IL-1beta, and IL-10 postoperatively^[31]^. Regarding neuronal function, NCD has been associated with elevated neuron specific enolase and tau, which are markers of brain injury and axonal injury, respectively^[11]^. CRP and IL-6 are more robustly increased among patients under 70 years of age *vs.* more elderly counterparts, and a recent study found a greater NCD in the younger cohort^[5]^. However, other studies have reported that elderly may be more susceptible to NCD generally, with one potential mechanism being an increased baseline blood brain barrier disruption^[32,33]^. It could therefore be interpreted that NCD is a more common complication amongst older patients, but the magnitude of NCD and its associated systemic inflammation is larger among younger patients when it does occur^[5]^. The role of intraoperative corticosteroids as an intervention to modulate inflammation and thus diminish postoperative cognitive dysfunction have been investigated with mixed results^[34,35]^.

It is important to briefly mention the close relationship between NCD and postoperative delirium. Both represent cognitive complications after major surgery, can present with similar symptoms to patients and their families, and have been tied to inflammatory processes^[36]^. Delirium involves a change in consciousness and attentional abilities often screened for using the Confusion Assessment Model for ICU (CAM-ICU) which takes a few minutes to administer at the bedside. At our institution this is part of the ICU nursing protocol. In contrast, NCD is almost exclusively diagnosed in the setting of research studies because it requires administering a battery of neuropsychological tests that assess multiple components of cognition^[37]^.

### Limitations

This study was limited by a small sample size (n=87) and enrollment from a single academic institution. Intraoperative MAP alone cannot determine cerebral perfusion, which also requires consideration of previous hypertensive injury to brain vasculature. Additionally, the method of obtaining an accurate picture of preoperative SBP was a subjective decision given available data. The number of recorded blood pressures varied widely between patients depending on how many available visits were recorded in our electronic medical record.

Although there are now protocols for both induction and maintenance of anesthesia at our institution, at the time this study began enrolling patients there was greater variability. It is possible that this contributes to cognitive outcomes and is not accounted for in these results. Additionally, it is not standard practice at our institution to use cerebral oximetry for all cases. Given that this is a retrospective study, we are unable to obtain or control that variable. It relied on previous outpatient blood pressure readings, which were not collected at the same time points for this study and may still have been influenced by “white coat hypertension”. The responses to RBANS follow-up at one month after surgery was limited to only 59 out of 87 patients which limits the ability to discuss long-term outcomes. Additionally, only a small subset of patients had sufficient blood samples to perform high-quality genomic analysis. The small sample size increases the weight of confounding factors. A larger sample size is needed to further investigate the role of inflammatory pathways. It is also important to note that although this study focused on preoperative chronic hypertension and perioperative blood pressure, there are numerous other factors that may influence postoperative neurocognitive outcomes including: hemodilution, non-pulsatile blood flow, potential for air or particulate embolization, and duration of time on CPB.

### Future Directions

This pilot study should be replicated as a multi-centered analysis with uniformly collected preoperative blood pressures, homogeny of procedures and urgency, diversity of patient population, and accurate measures of intraoperative cerebral perfusion. Diastolic blood pressure, corrected for factors such as aortic regurgitation, should be included. Additionally, future research could investigate other risk factors associated with the mechanism of NCD both between and within hypertensive and normotensive patients. It is also important to distinguish the contribution of each component of cardiac surgery to NCD (*i.e.*, surgical trauma, anesthetics, etc.). Furthermore, investigation of recovery at one month, followed by assessment of cognitive function chronically could link risk factors to NCD recovery and long-term cognitive decline as well as NCD itself. These studies provide a detailed pathophysiologic explanation of a surgical phenomenon, and contribute to a framework for identifying, quantifying, and seeking to improve modifiable risk factors.

## CONCLUSION

This study proposes that high SBP preoperatively is a risk factor for NCD after cardiac surgery using CPB. It also proposes that preoperative hypertension contributes to one component of the pathogenesis of NCD: chronic exposure to high blood pressure harbors preoperative neuroinflammation that leaves patients more vulnerable to NCD via reduced ability to attenuate oxidative stress, reduced ability to attenuate inflammation, and reduced neuronal protection. Preoperative neuroinflammation may be caused by hypertension’s contribution to a disrupted blood-brain-barrier, the renin-angiotensin-aldosterone-system’s contribution to reactive oxygen species, and pro-inflammatory cytokines (tumor necrosis factor alpha, IL-1beta, IL-6) in the presence of decreased IL-10 mRNA by angiotensin II^[32,33]^. Hypertension also contributes to cerebral atherosclerosis, likely increasing the risk of micro-embolisms and cerebral ischemia. As mentioned before, altered autoregulation and cerebral perfusion during cardiac surgery and CPB likely plays an additional role in the differential effects of surgery on normotensive and hypertensive patients, but this was not examined in the current study. This proposed mechanism is one component of the complex pathophysiology of NCD.
